# An Organic Acid Based Counter Selection System for Cyanobacteria

**DOI:** 10.1371/journal.pone.0076594

**Published:** 2013-10-01

**Authors:** Matthew B. Begemann, Erin K. Zess, Eric M. Walters, Emily F. Schmitt, Andrew L. Markley, Brian F. Pfleger

**Affiliations:** 1 Microbiology Doctoral Training Program, University of Wisconsin-Madison, Madison, Wisconsin, United States of America; 2 Department of Chemical and Biological Engineering, University of Wisconsin-Madison, Madison, Wisconsin, United States of America; University of Houston, United States of America

## Abstract

Cyanobacteria are valuable organisms for studying the physiology of photosynthesis and carbon fixation, as well as metabolic engineering for the production of fuels and chemicals. This work describes a novel counter selection method for the cyanobacterium *Synechococcus* sp. PCC 7002 based on organic acid toxicity. The organic acids acrylate, 3-hydroxypropionate, and propionate were shown to be inhibitory towards *Synechococcus* sp. PCC 7002 and other cyanobacteria at low concentrations. Inhibition was overcome by a loss of function mutation in the gene *acsA*, which is annotated as an acetyl-CoA ligase. Loss of AcsA function was used as a basis for an acrylate counter selection method. DNA fragments of interest were inserted into the *acsA* locus and strains harboring the insertion were isolated on selective medium containing acrylate. This methodology was also used to introduce DNA fragments into a pseudogene, *glpK*. Application of this method will allow for more advanced genetics and engineering studies in *Synechococcus* sp. PCC 7002 including the construction of markerless gene deletions and insertions. The acrylate counter-selection could be applied to other cyanobacterial species where AcsA activity confers acrylate sensitivity (e.g. *Synechocystis* sp. PCC 6803).

## Introduction

Current research concerning the physiology of cyanobacteria has led to an increased understanding of photosynthesis, CO_2_ fixation, and metabolism [1–3]. In addition to basic science, researchers are also exploring the application of cyanobacteria for the production of biofuels and chemicals [4,5]. Continued advances in these areas will require more efficient methods for genetic manipulation and gene expression [6,7]. In particular, counter selection methods that are applicable to multiple strains of cyanobacteria would be useful in dealing with the issues of multiple chromosomes and limited antibiotic resistance cassettes. Currently, a *sacB* counter selection system was demonstrated in a glucose tolerant strain of *Synechocystis* sp. PCC 6803 (PCC 6803) and a *mazF* based system in wild type PCC 6803 [8,9]. Unfortunately, attempts to build a *sacB* counter selection system have been unsuccessful and *mazF* has not been demonstrated in *Synechococcus* sp. PCC 7002 (PCC 7002), another model organism that grows rapidly and is widely tolerant to light intensity, temperature and salt conditions. Due to these advantages, PCC 7002 has recently been explored for the production of α-olefins and sugars via metabolic engineering [10,11].

Since a counter selection system was not available for PCC 7002, we developed a new system based on sensitivity to organic acids. In general, organic acids can cause toxicity by depleting proton motive force, accumulating anions, and inhibiting enzymes [12–14]. The organic acids of interest in this work are acrylate, 3-hydroxypropionate, and propionate. Acrylate was shown to inhibit beta oxidation in *Pseudomonas* and *Ralstonia* species, 3-hydroxypropionate was shown to inhibit the chorismate pathway in *Escherichia coli*, and propionate was shown to inhibit gluconeogenesis via metabolism to 2-methylcitrate in *Salmonella* [15–18]. Additionally, exposure to propionate was shown to cause a decrease in the free Coenzyme-A (CoA) pool in a species of *Synechococcus* [19].

This work demonstrates that these three organic acids, acrylate, 3-hydroxypropionate, and propionate, are inhibitory to multiple species of cyanobacteria at low concentrations. This inhibition can be overcome in PCC 7002 by a single loss of function mutation in a gene encoding a CoA-ligase (*acsA*). These observations were used as a basis for the development of a counter selection system using acrylate sensitivity. Acrylate was chosen amongst the organic acids due to its inhibitory activity in multiple species and dramatically increased tolerance in strains without AcsA activity. This counter selection method was used to directly introduce and select for integrations into the *acsA* locus, as well as generate a scarless integration in a neutral locus. The ability to rapidly introduce heterologous gene constructs and make markerless deletions will advance both basic science and applied research efforts in cyanobacteria.

## Materials and Methods

### Chemicals and 3-hydroxypropionate quantification

Acrylate was purchased from MP Biomedicals (ICN211387). 3-hydroxypropionate was purchased from TCI America (H0297) as a ca. 3.6 mol/L solution in water. Additional chemicals and reagents were purchased from Fisher Scientific and Sigma Aldrich. The 3-hydroxypropionate solution purchased from TCI had an unknown purity, so the solution was quantified with an enzyme assay using propionyl-CoA synthase. *Escherichia coli* (*E. coli*) BL21 harboring pKS1 (expressing propionyl-CoA synthase) was grown in lysogeny broth (LB) to an OD_600_ of 0.4 and induced with 0.4 mM isopropyl β-D-1-thiogalactopyranoside (IPTG). The induced culture was incubated at 30 °C for 4 h before centrifugation. The resulting cell pellet was resuspended in twice the volume of 100 mM Tris pH 7.8 and lysed by sonication. The cell debris was removed by centrifugation at 16,000 x g for 10 min at 4 °C. The supernatant was incubated at 63 °C for 10 min and the precipitated protein was removed by centrifugation at 20,000 x g. The resulting protein fraction was concentrated 2X using an Amicon Ultra-4 centrifugation column to increase activity. The resulting enzyme was used in an assay to quantify 3-HP by NAPH reduction as described in Schneider et al [20].

### Strains, culturing conditions, and media

**Figure 1:**
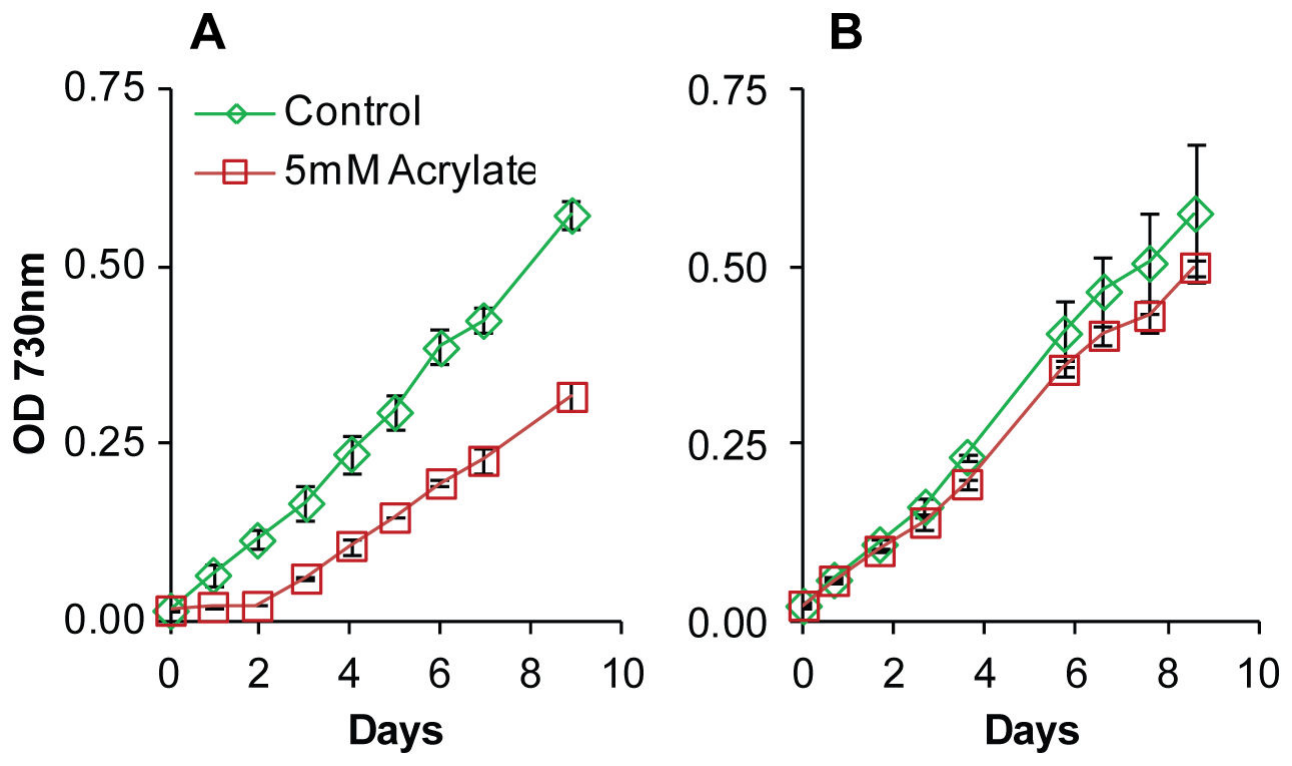
Growth of wild type and an adapted strain of PCC 7002 with acrylate. (A) Growth of wild type PCC 7002 in unmodified medium (green diamonds) or 5 mM acrylate (red squares). (B) Growth of acrylate adapted cultures of PCC 7002 in unmodified medium (green diamonds) or 5 mM acrylate (red squares). Data points are the mean of biological triplicates and error bars represent the standard deviation.

All strains used in this study are listed in Table 1. PCC 7002, *Synechococcus* sp. PCC 7942 (PCC 7942), and PCC 6803 were obtained from the Pasteur Culture Collection. PCC 7002 was grown in Medium A^+^ pH 8.0 [21]. PCC 7942 and PCC 6803 were grown in BG-11 [22]. Unless otherwise noted, liquid cultures were grown in 10 mL volumes at a light intensity of 140 µE/m^2^/s at 35 °C and bubbled with air. Agar plates were incubated under the same light and temperature conditions. Cultures used to observe the initial mutant population (Figure 1) and harvest RNA were grown in 50 mL volumes without agitation or supplementation with air. Cultures were inoculated using patches grown from a single colony on solid media under the same conditions. Unless otherwise noted, cell growth was monitored by measuring the optical density at 730nm (OD_730_) using a Spectrophotometer 20 (Milton Roy).

**Table 1:** Cyanobacterial strains used in this study.

**Strain**	**Genotype**	**Comments**
BPSyn_001	*Synechococcus* sp. PCC 7002	Obtained from the Pasteur Culture Collection
BPSyn_006	*acsA*::*aadA*	Replacement of *acsA* with streptomycin resistance marker
BPSyn_014	*acsA*::BC	Replacement of *acsA* with a 40 bp bar code
BPSyn_015	*acsA*::p*cpcB*_YFP	Replacement of *acsA* with YFP under a constitutive promoter
BPSyn_022	*acsA*::BC *glpK::acsA aadA*	Replacement of *glpK* of BPSyn014 with *acsA* and a streptomycin resistance marker
BPSyn_026	*acsA*::BC *glpK::acsA*W49L *aadA*	Replacement of *glpK* of BPSyn014 with *acsA*W49L and a streptomycin resistance marker
BPSyn_027	*acsA*::BC *glpK::*p*cpcB*_YFP	Replacement of *glpK* of BPSyn014 with YFP under a constitutive promoter
BP6803_001	*Synechocystis* sp. PCC 6803	Obtained from the Pasteur Culture Collection
BP6803_002	*sll0542*::KmR	Replacement of *sll0542* with kanamycin resistance marker
BP7942_001	*Synechococcus* sp. PCC 7942	Obtained from the Pasteur Culture Collection

### Determining minimum inhibitory concentrations

Inocula were prepared by growing cultures of wild type and mutant cyanobacteria to an OD_730_ of 0.3. These cultures were used to inoculate, to an initial OD_730_ of 0.03, a 96 well plate containing buffered media with increased concentrations of the compound of interest. Stocks of the compounds of interest were prepared in Medium A^+^ and adjusted to a pH of 8.0. Wild type PCC 7002 was challenged with organic acid concentration increments of 5 µM for acrylate and 1 mM for 3-hydroxypropionate and propionate. Mutant strains with increased tolerance were further challenged with concentration increments of 10 mM acrylate, 5 mM 3-hydroxypropionate, and 100 mM propionate. Plates were incubated at 35 °C at a light intensity of 140 µE/m^2^/s. OD_730_ was measured periodically using a plate reader (Tecan M1000). The minimum inhibitory concentration (MIC) was determined as the concentration at which no increase in OD_730_ was observed after 48 h.

### Quantification of mutation frequency

Wild type PCC 7002 was grown to an OD_730_ of 1.0 and serially diluted in triplicate on both unmodified and acrylate-containing plates. Acrylate concentrations of 50 µM, 500 µM, 5 mM, and 50 mM were used. Plates were incubated for 5 days and counted for colonies. Mutation frequency is defined as the number of acrylate tolerant colony forming units, divided by the total number of colony forming units.

### RNA-sequencing and analysis

Cultures of PCC 7002 were grown in 5 mM acrylate or unmodified medium A^+^ in quadruplet to an OD_730_ of 0.5-0.6. Duplicate cultures from each condition were harvested for RNA sequencing. RNA was isolated from PCC 7002 using the Trizol 95 method and a RNeasy kit (Qiagen) [23]. RNA purity was verified using an Agilent RNA 600 Nano Kit and Agilent 2100 Bioanalyzer. Ribosomal RNA was removed using the Epicentre rRNA removal kit for Gram negative bacteria (RZNB1056). RNA was sequenced by the University of Wisconsin Gene Expression Center using an Illumina HiSeq 2000. The resulting RNA sequencing reads were aligned to the PCC 7002 genome (Genbank ID CP000951.1-CP000957.1) and analyzed using CLC Genomics Workbench software. Reads per kilobase per million total reads (RPKM) was used to quantify expression values [24]. Genes identified as being differentially regulated were defined as having greater than 2 fold changes in RPKM and a P-value less than 0.05. Single nucleotide polymorphisms (SNPs) were identified using the CLC Genomics Workbench software. Genes of interest were identified as having SNPs in acrylate cultures but not control cultures. These data have been deposited in NCBI’s Gene Expression Omnibus (REF) and are accessible through the GEO Series accession number GSE48981 (http://www.ncbi.nlm.nih.gov/geo/query/acc.cgi?acc=GSE48981).

### Mutant construction

All plasmids used in this study are listed in Table 2. A PCC 7002 Δ*acsA* mutant (BPSyn_006) was constructed by double homologous recombination as previously described [25]. Briefly, a linear DNA fragment was constructed containing a streptomycin resistance marker derived from pSRA81 (*aadA*) flanked by approximately 600 bp regions that are homologous to regions directly 5’ and 3’ of the *acsA* (SYNPCC7002_A1838) gene. The *acsA* upstream and *acsA* downstream pieces were individually amplified using the primers in Table 3, digested, and then ligated together with the digestion fragment of pSRA81. The resulting fragment was PCR amplified and transformed into wild type PCC 7002. A similar protocol was used to replace *acsA* with a 40 bp barcode sequence, resulting in strain BPSyn_014. A linear fragment was created with 600 bp homologous regions and a 40 bp barcode sequence in between. This fragment was transformed into PCC 7002 and mutants were selected on plates containing 50 µM acrylate. Positive clones were streaked out on plates containing 10 mM acrylate to achieve complete chromosomal segregation. The resulting mutant had a deletion of *acsA* without a residual antibiotic resistance cassette. Complementation of *acsA* was performed through the introduction of *acsA* under the native *acsA* promoter into *glpK* (SYNPCC7002_A2842). Due to a frameshift mutation, *glpK* is a pseudogene in PCC 7002. For this reason, *glpK* was chosen as a neutral insertion site. Plasmids pGLPK_acsA_Sp^R^ and pGLPK_acsAW49L_Sp^R^ were constructed with homologous flanking regions to replace *glpK* with either (1) a wild type copy of *acsA* or (2) *acsA*W49L under the native *acsA* promoter along with a streptomycin resistance marker, using the *in vitro* recombination method using primers in Table 3 [26]. Plasmids pACSA_pcpcB_YFP and pGLPK_pcpcB_YFP were constructed in a similar manner using the *cpcB* promoter from PCC 6803 and yellow florescent protein (YFP) gene from plasmid pAQ1_Exp_YFP built by Xu et al. [27]. All cyanobacterial strains were screened by colony PCR for proper integration and full segregation of all chromosomes.

**Table 2:** Plasmids used in this study.

**Plasmid**	**Genotype**	**Comments**
pET28b_acsA	P_T7_ *acsA* Km^R^	Cloned *acsA* into the MCS of pet28b for His-tag purification
pET28b_acsAW49L	P_T7_ *acsA*W49L Km^R^	Cloned *acsAW49L* into the MCS of pet28b for His-tag purification
pGLPK_acsA_Sp^R^	P_*acsA*_ *acsA* Sp^R^	Replaced the flanking regions of pAQ1-Exp-PcpcBYFP (Xu et al) with 500 bp flanking regions of *glpK*
pGLPK_acsAW49L_Sp^R^	P_*acsA*_ *acsA*W49L Sp^R^	Used Quick Change PCR to make the W49L mutation in pGLPK_acsA_Sp^R^
pACSA_pcpcB_YFP	P_*cpcB*_YFP	Replaced the flanking regions of pAQ1-Exp-PcpcBYFP (Xu et al) with 500 bp flanking regions of *acsA*
pGLPK_pcpcB_YFP	P_*cpcB*_YFP	Replaced the insert of pGLPK_acsA_Sp^R^ with YFP

**Table 3:** Oligonucleotides used in this study.

**Primer Name**	**Sequence (5'–3')**
Construction of BPSyn_006	
acsA-a1	ATGATCATCGGGGAATGCTCTTGATTC
acsA-a2	AAAACTGCAGTCGTGGGATTTATTTCACCCCATTGTC
acsA-b1	AAAAGGATCC GTCTTAATGTATGAAGGCGCACCC
acsA-b2	CCTCTGGACATCTCCCTCAAGG
Construction of BPSyn_014	
acsA UpF	GATTTTCAAGCCCAGGTG
acsA UpR-BC	CCCGCATGCCCGGTCTACCTGTACACGAGTTCGTTTCAATGAAGGCGAAAC
acsA DwnF-BC	CCCGCATGCCTTCGTAATAAGGATGCGCTCAAGTTGATCGAATGTATG
acsA DwnR	CTATATCTGGCAAACAACTTTGGC
acsA Amplify F (A1)	AGGTGACTGCCGCACTCA
acsA Amplify R (A2)	CTGGCAAACAACTTTGGCTGCC
internal acsA F (B1)	ATTTTTGCGCCAGGTTTTGAG
acsA screen R (B2, C2)	CCGTAACCTCCTAGGATTGGG
ΔacsA BC F (C1)	AACTGTGTACAGGTAGACCG
Construction of pGLPK_acsA_SpR	
glpKUp Fwd (E1)	TGAAGCGATTGGCTATGATCTACCAAAG
glpKUp Rev	CTCAGGGAACCATAAGAATTCTTTTTTAAATGGGTTAAATTAGGTC
pacsA Fwd (G1)	GAATTCTTATGGTTCCCTGAGGCGATC
T7 Rev (G2)	CAAAAAACCCGTCAAGACCCGTTTAG
glpKDwn Fwd	GGGTCTTGACGGGTTTTTTGTTACTGCTCCATGACCAACATTATTCCC
glpKDwn Rev (E2)	GAAACGAGATTATCTAAAACAGAAGCATGG
pGLPK Fwd	GTTTTAGATAATCTCGTTTCGCATGCGACGTCGGGCCC
pGLPK Rev	GATCATAGCCAATCGCTTCAATGCATAGCTTGAGTATTCTATAGTGTCACC
Construction of pGLPK_acsAW49L_SpR	
acsAW49L QC Forward	AAAATGACCCCGAAGGCTTTTTGGGGGAACTCG
acsAW49L QC Reverse	CGAGTTCCCCCAAAAAGCCTTCGGGGTCATTTT
Construction of pET28b_acsA	
NdeIacsAForward	AAAAAACATATGTCCGAACAAAACATTGAATCCATCCTC
BamHIacsAReverse	AAAAAAGGATCCTTAGCCCCGGAGTTGATCGAGGA
Construction of pGLPK_pcpcB_YFP	
cpcBFwdGib	GAATTCGTTATAAAATAAACTTAACAAATCTATACCC
T7RevGib	CAAAAAACCCGTCAAGACCCGTTTAG
glpKUp Rev	CTCAGGGAACCATAAGAATTCTTTTTTAAATGGGTTAAATTAGGTC
glpKDwn Fwd	GGGTCTTGACGGGTTTTTTGTTACTGCTCCATGACCAACATTATTCCC
YFP Screen Fwd (D1)	GACGACGGCAACTACAAGAC
YFP Screen Rev (D2)	GGTGTTCTGCTGGTAGTG GT
glpK internal Fwd (F1)	CACCGCTGGGGCTTGTATCC
glpK outside Rev (F2)	GAGAGA AAGGCTTCATGATCAAGGG
Construction of pACSA_pcpcB_YFP	
cpcBFwdGib	GAATTCGTTATAAAATAAACTTAACAAATCTATACCC
pAQ1 into pACSA Rev	CATACATTCGATCAACTTGAGCGCTCTAGACAAAAAACCCGTCAAG
acsA Down Fwd	GCGCTCAAGTTGATCGAATGTATG
acsA Up Rev	GTTCGTTTCAATGAAGGCGAAAC

### AcsA activity assay

Wildtype *acsA* and the *acsA*W49L mutant were cloned into the pET-28a plasmid (Novagen) with an N-terminal His-tag. Strains of *E. coli* DH10B harboring these plasmids were grown in 50 mL LB at 37 °C to an OD_600_ of 0.6-0.8 and induced with 1 mM IPTG. Induced cultures were incubated at 37 °C for 3 h. The cultures were centrifuged at 3,000 x g for 15 min and the resulting cell pellets were frozen at -20 °C. BugBuster reagent (Novagen) was used to lyse cells and collect the soluble protein fraction. AcsA protein was purified from the soluble fraction using Ni-NTA His-tag affinity chromatography. Reactions containing 500 nM enzyme and 2 mM organic acid were performed at 37 °C for 6 min. Specific activity was determined by measuring the loss of free Coenzyme A over time using Ellman’s reagent. Specific activity is defined as µmol free CoA min^-1^ µmol^-1^ AcsA. 3-hydroxypropionate is abbreviated to 3-HP.

### Counter selection

PCC 7002 was transformed by adding 3 µg of linearized plasmid to 2 mL of culture at OD_730_ 1-1.5. The cultures were incubated without agitation overnight under the previously mentioned light and temperature conditions. Transformants were selected by plating both 100 µL and the remaining 1.9 mL transformation suspension, concentrated, on solid media supplemented with 50 μM acrylate. Plates were incubated in the light for 2-3 days at 35 °C. A successful transformation usually had significantly more colonies than the no-DNA, negative control. Single colonies were patched onto solid media supplemented with 50 μM acrylate. This intermediate step was used to avoid colony PCR contamination by excess DNA used in the transformation. After 1 day of growth at standard conditions, the presence of the insertion was confirmed by colony PCR. Single colonies of positive transformants were isolated on solid media containing 10 mM acrylate. Incubation for 3-5 days allowed for complete segregation of the transformants. Segregation was confirmed by colony PCR, using primers flanking the insertion site, specific to the insertion sequence, and specific to the sequence being replaced. Confirmed mutants were maintained on unmodified medium.

### Florescence Detection

Cultures of PCC 7002 containing YFP were grown in triplicate to an OD_730_ 0.5-1. Cultures were normalized to 4 OD_730_ * mL and centrifuged at 5,000 x g for 10 min. The resulting pellets were aspirated, resuspended in 300 µL BugBuster Protein Extraction Reagent (Novagen), rocked at room temperature for 30 min, and centrifuged at 16,000 x g for 25 min at 4 °C. The florescence of the resulting supernatant was measured (excitation 514 nm, emission 527 nm) using a Tecan M1000 plate reader.

### Quantification of acetate

After 75 h of growth, 1 mL samples taken from triplicate cultures were centrifuged at 16,000 x g for 10 min. Each supernatant was passed through a 0.22 µm filter prior to analysis via gas chromatography and flame ionization detection using a Shimadzu GC-2010 oven with a Restek Stabilwax-DA fused silica column (11052) and AOC-20i auto-injector. The limit of detection for acetate was 10 µM.

## Results and Discussion

### Cyanobacteria are sensitive to several organic acids

Two components are necessary for a successful counter selection method. First, a compound must be identified that is inhibitory at low concentrations. Second, this inhibition must be removed by the loss of a genetic element. Here, the toxicity, as judged by MIC, of organic acids towards PCC 7002 PCC 6803, and PCC 7942 was assessed by exposing each strain to increasing concentrations of organic acids in buffered media. The organic acids acrylate, 3-hydroxypropionate, and propionate were each inhibitory to all three cyanobacteria at relatively low concentrations (Table 4). Acrylate generated the lowest MIC for each strain (<50 µM) followed by propionate (0.25-4 mM) and 3-hydroxypropionate (2-70 mM).

**Table 4:** Minimum inhibitory concentration (MIC) of organic acids.

**Strain**	**acrylate (mM**)	**3-HP (mM**)	**Propionate (mM)**
*Synechococcus* sp. PCC 7942	0.003	2	0.25
*Synechocystis* sp. PCC 6803	0.05	70	0.25
BP6803_002 (*sll0542*::KmR)	70	70	No Data
*Synechococcus* sp. PCC 7002	0.025	10	4
BPSyn_006 (*acsA*::*aadA*)	70	260	>400
BPSyn_014 (*acsA*::BC)	70	260	No Data
BPSyn_022 (*acsA*::BC *glpK*::*acsA aadA*)	0.01	15	No Data
BPSyn_026 (*acsA*::BC *glpK*::*acsA*W49L *aadA*)	7	No Data	No Data

### Mutations increase organic acid tolerance

Wild type PCC 7002 was inoculated into medium containing 5 mM acrylate, a concentration several orders of magnitude above the MIC. The OD_730_ was observed relative to control cultures for a period of 200 h. For the first 50 h of incubation, no increase in OD_730_ was observed in acrylate containing cultures. After this long lag the OD_730_ increased at rate similar to the control culture (Figure 1A). Sub-culturing of the acrylate exposed cultures into fresh medium containing 5 mM acrylate resulted in no growth inhibition (Figure 1B). Additionally, acrylate exposed cultures that were serially sub-cultured in medium without acrylate for four iterations maintained the ability to grow in an acrylate concentration above the wild type MIC. These data suggested that a mutation arose within the population that resulted in increased tolerance to acrylate. When wild type PCC 7002 was plated onto solid media containing 5 mM acrylate, mutant colonies were observed at a frequency of around 10^-6^. Mutants were observed on plates containing 500 µM and 50 µM acrylate at 5 and 10 times the frequency of 5 mM, respectively. No mutants were observed on plates containing 50 mM acrylate. This rate of mutation frequency was suggestive of a loss of function mutation [28]. Additionally, the correlation between mutation frequency and concentration of acrylate suggested that gene dosage or the presence of partial loss of function mutations existed. Interestingly, mutants obtained on acrylate containing medium were also more tolerant of 3-hydroxpropionate and propionate.

### RNA-Sequencing was used to identify mutations

RNA was harvested from the cultures described in Figure 1A. Samples were processed and sequenced to identify changes in gene expression caused by exposure of wild type PCC 7002 to acrylate above the MIC. The transcriptome of cultures grown with acrylate had significantly reduced transcript levels for genes associated with oxidative stress (Table 5) compared to cells grown in unsupplemented media. In particular, *isiA* and *sufA* were down-regulated >10- and >2-fold, respectively. This was not surprising, as acrylate was shown to act as an antioxidant in eukaryotic algae [29]. Due to the starting OD_730_ (0.03) and the OD_730_ at which the cultures were harvested (0.5), it was assumed that the majority of each population carried a mutation for acrylate tolerance. An analysis of single nucleotide polymorphisms (SNPs) relative to the published genome sequence (GenBank CP000951.1) was performed (Table 6). Four genes were identified with SNPs in all acrylate samples but not present in any of the control samples. The gene *acsA* (GenBank NC_010475.1) was of interest because some acetyl-CoA ligases have been shown to have activity towards acrylate [30]. In acrylate exposed samples, the reads spanning bp 152 of acsA, 60% (32/53) had a point mutation of a G to a T. The point mutation results in an amino acid change of Trp49 to Leu. This amino acid is part of a conserved region in the *acsA* of PCC 7002, PCC 6803 (GenBank AP012278.1) and *Escherichia coli* K12 (GenBank NP_418493.1), suggesting it is integral to a functional protein. These results led to the hypothesis that loss of function of *acsA* would result in the observed increase in organic acid tolerance.

**Table 5:** Genes differentially expressed in the presence of 5 mM acrylate.

**Feature ID**	**Annotation**	**Fold Change**	**P-value**
hliA	high light/nutrient deprived stress response	5.2	9.75E-03
SYNPCC7002_A1476	high light inducible	3.46	1.67E-05
nirA	nitrite reductase	3.42	0
SYNPCC7002_A2493	conserved hypothetical protein	3.37	1.13E-04
narK	nitrate transporter	3.33	0
narB	nitrate reductase	3.29	2.00E-15
SYNPCC7002_G0056	hypothetical protein	3.09	0.03
SYNPCC7002_A1237	hypothetical protein	2.97	1.67E-05
sigC	group II sigma-70 type sigma factor	2.89	2.75E-12
SYNPCC7002_A0782	conserved hypothetical membrane protein	2.89	0
SYNPCC7002_G0109	hypothetical protein	2.79	0
SYNPCC7002_A1733	ABC 3 transport family protein (Mn^2+^)	2.78	2.44E-15
rubA	rubredoxin (photosynthesis lipid)	2.77	0
SYNPCC7002_A2433	galactosyl-1-phosphate transferase	2.68	0.06
SYNPCC7002_A2086	conserved hypothetical protein	2.66	0.02
rpaB	two-component response regulator	2.64	8.39E-11
SYNPCC7002_A2692	hypothetical protein	2.6	6.89E-03
ndhL	NADH dehydrogenase subunit L	2.58	8.58E-11
SYNPCC7002_A2772	conserved hypothetical	2.55	0.01
SYNPCC7002_A0125	formate/nitrite transporter	2.53	7.38E-12
SYNPCC7002_A1238	predicted ATPase/GTPase	2.53	2.22E-16
SYNPCC7002_A0472	conserved hypothetical protein	2.53	8.69E-04
SYNPCC7002_A2240	CobQ/CobB nucleotide binding protein	2.51	1.02E-13
SYNPCC7002_G0125	hypothetical protein	2.39	1.97E-04
nblA	putative phycobilisome degradation protein	2.37	1.78E-05
SYNPCC7002_A0468	bacterial domain of unknown function	2.33	8.85E-06
SYNPCC7002_A0793	AhpC/TSA family protein	2.29	0
hliA	high light/nutrient deprived stress response	2.28	8.32E-03
SYNPCC7002_A1618	glycosyl transferase family	2.26	7.11E-14
rplE	50S ribosomal protein L5	2.24	0
SYNPCC7002_A2607	probable Rieske iron-sulfur protein	2.24	6.43E-04
SYNPCC7002_A1619	conserved hypothetical protein	2.21	6.11E-14
SYNPCC7002_A2241	conserved hypothetical protein	2.21	0.03
SYNPCC7002_D0026	conserved hypothetical protein	2.19	3.90E-04
SYNPCC7002_A0470	sodium-dependent bicarbonate transporter	2.19	3.24E-06
cupA	CO2 hydration protein	2.19	3.66E-08
SYNPCC7002_A2535	conserved hypothetical protein	2.18	2.30E-05
SYNPCC7002_A1908	conserved hypothetical protein	2.14	0.02
SYNPCC7002_A0429	conserved hypothetical protein	2.14	3.23E-03
SYNPCC7002_A0397	ABC transporter	2.14	4.62E-06
SYNPCC7002_F0033	hypothetical protein	2.13	1.24E-13
SYNPCC7002_A0469	conserved hypothetical protein	2.1	3.40E-06
SYNPCC7002_A0467	putative cysteine protease superfamily	2.08	2.47E-08
rplP	ribosomal protein L16	2.07	1.58E-11
hslO	Hsp33-like chaperonin	2.05	0
SYNPCC7002_A2244	conserved hypothetical protein	2.05	7.33E-08
rplN	ribosomal protein L14	2.04	0
SYNPCC7002_C0016	hypothetical protein	2.04	5.35E-06
SYNPCC7002_A0582	precorrin isomerase	2.03	0
SYNPCC7002_A0175	conserved hypothetical protein	2.03	3.26E-08
SYNPCC7002_A0421	ABC-type transport protein	2.03	7.64E-03
rplR	ribosomal protein L18	2.03	0
rdgB	non-canonical purine NTP pyrophosphatase	2.03	6.86E-14
SYNPCC7002_A0395	ABC transporter	2.02	9.73E-10
SYNPCC7002_A1477	conserved hypothetical protein	2.02	0
SYNPCC7002_A0396	ABC transporter	2.02	3.88E-04
SYNPCC7002_A0102	hypothetical protein	2.01	5.70E-05
SYNPCC7002_A0381	conserved hypothetical protein	2.01	4.38E-11
SYNPCC7002_A1368	conserved hypothetical protein	2.01	0
isiA	photosystem II chlorophyll-binding protein	-14.42	6.16E-04
SYNPCC7002_A1291	flavodoxin	-8.27	6.13E-04
SYNPCC7002_G0019	siderophore biosynthesis protein	-8.12	7.80E-06
SYNPCC7002_G0018	esterase/lipase	-7.21	7.75E-07
SYNPCC7002_G0083	ABC transporter	-6.17	6.41E-04
SYNPCC7002_A2659	conserved hypothetical	-5.05	4.01E-03
SYNPCC7002_G0021	siderophore biosynthesis protein	-4.48	0.02
SYNPCC7002_A2660	conserved hypothetical	-4.39	0.02
fecC	FecCD transport (permease) family	-4.37	0.02
SYNPCC7002_G0079	ABC transporter, permease, FecCD family	-4.1	2.08E-03
SYNPCC7002_G0090	TonB family C-terminal domain protein	-4.08	0.03
SYNPCC7002_A0933	hypothetical protein	-4.07	2.07E-37
SYNPCC7002_G0089	MORN repeat protein	-4.02	0.01
SYNPCC7002_G0086	ATP-binding protein of ABC transporter	-4.01	7.98E-03
SYNPCC7002_G0082	glyoxalase family protein family	-4	3.64E-03
SYNPCC7002_G0022	siderophore biosynthesis protein	-3.78	0.03
SYNPCC7002_A2657	hypothetical protein	-3.09	0.02
fecD	FecCD transport family	-3.06	1.24E-03
SYNPCC7002_G0017	hypothetical protein	-2.97	0.03
sufA	iron transport protein	-2.96	0.02
SYNPCC7002_A0612	tRNA-Arg	-2.9	5.05E-03
SYNPCC7002_G0023	siderophore biosynthesis protein	-2.85	0.06
SYNPCC7002_A0871	ABC transporter	-2.78	0.04
SYNPCC7002_G0084	conserved hypothetical protein	-2.78	0.02
SYNPCC7002_E0003	hypothetical protein	-2.7	1.35E-72
ardA	antirestriction protein	-2.45	1.65E-19
SYNPCC7002_G0080	iron compound ABC transporter	-2.41	3.26E-04
SYNPCC7002_A1858	conserved hypothetical	-2.4	2.41E-04
SYNPCC7002_E0004	hypothetical protein	-2.35	1.16E-39
SYNPCC7002_A0934	hypothetical protein	-2.32	2.30E-03
SYNPCC7002_A2658	oxidoreductase	-2.07	0.02
nrdA	ribonucleotide reductase subunit alpha	-2.01	5.95E-04
SYNPCC7002_A2117	conserved hypothetical protein	-2.01	2.34E-03

**Table 6:** Genes identified in SNP analysis.

**Gene**	**Annotation**	**Base Change**	**Frequency (%)^a^**	**Coverage^b^**	**Amino Acid Change**
acsA	acetate-CoA ligase	G/T	60	53	Trp49Leu
SYNPCC7002_A0421	ABC-type transport protein	C/T	43	65	Arg186Cys
SYNPCC7002_A2260	conserved hypothetical protein	C/A	65	37	Gln7Lys
SYNPCC7002_A2570	4Fe-4S binding domain protein	T/G	38	26	Val1Gly

a Percentage of reads with the specified mutation

b The total number of reads covering the specified base position

### AcsA has activity towards acrylate and other organic acids

**Figure 2:**
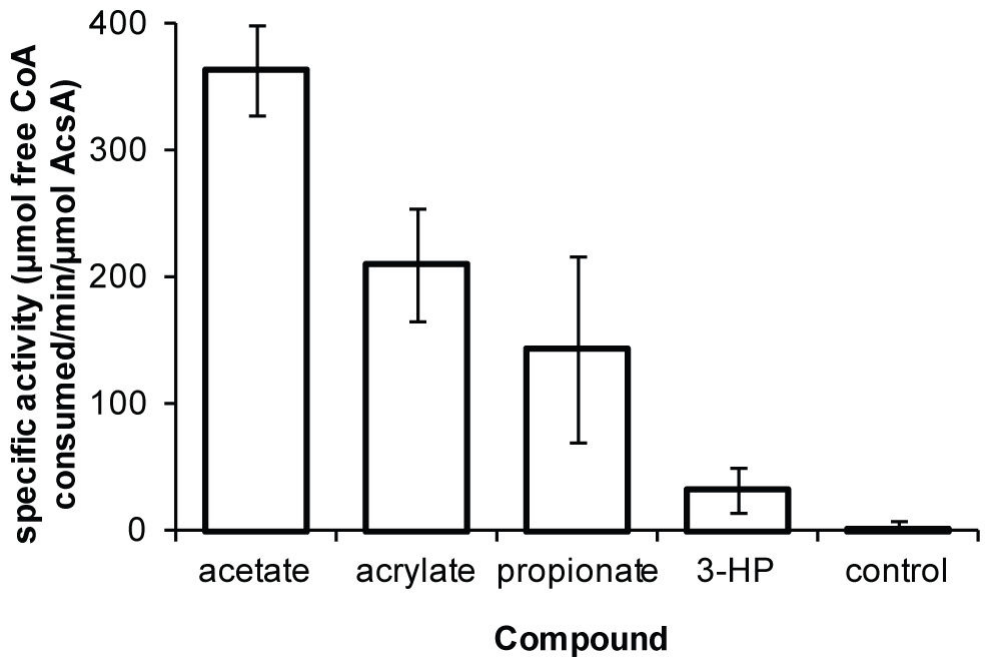
Specific activity of AcsA towards the organic acids acetate, propionate, acrylate, and 3HP.

To examine the function of AcsA, His-tagged versions of the wild type gene and an *acsA*W49L mutant were heterologously expressed in *E. coli*. The wild type AcsA protein was found in the soluble protein fraction and was purified using Ni-NTA His-tag affinity chromatography. Expression of the *acsA*W49L mutant gene generated an insoluble protein. Attempts to purify the AcsAW49L protein from the soluble fraction with Ni-NTA affinity chromatography failed. The activity of purified AcsA protein towards various organic acids was assessed *in vitro*. CoA ligase activity was observed for acetate, acrylate, 3-hydroxypropionate, and propionate (Figure 2). No activity was observed for succinate under these assay conditions (data not shown). The activity of AcsA towards these organic acids supports the results that a single mutation increases tolerance to all three compounds. While the presence of acyl-CoA thioesters may be toxic, we hypothesize that the observed toxicity of acrylate and other organic acids was caused because the CoA thioesters were metabolic dead-ends and the continuous activity of AcsA depleted the free CoA pool. This hypothesis is supported by prior work in which a species of *Synechococcus* grown in the presence of propionate was no longer able to import acetate and was observed to have decreased levels of free CoA [19].

### Loss of function of *acsA* increases organic acid tolerance

A PCC 7002 *acsA* deletion mutant, BPSyn_006, was constructed by double homologous recombination to replace *acsA* with the streptomycin resistance marker *aadA*. BPSyn_006 had dramatically higher MIC values for acrylate, 3-hydroxypropionate, and propionate (Table 4). Specifically, this mutant had 2,800-fold, 26-fold, and >100 fold increase in MIC to acrylate, 3-hydroxypropioante, and propionate, respectively. A similar observation was made in PCC 6803 by replacing *acsA* (sll0542) with a kanamycin resistance cassette, resulting in strain BP6803_002. BP6803_002 has a 3,500-fold increased tolerance to acrylate, but no increased tolerance to 3-hydroxypropionate. This suggests that the mechanism of toxicity of 3-hydroxypropionate is different in PCC 7002 and PCC 6803. This difference between species and the order or magnitude increase in acrylate tolerance for both species makes acrylate a more attractive compound for a universal counter selection.

### The organic acid tolerance phenotype was complemented in PCC 7002

**Figure 3:**
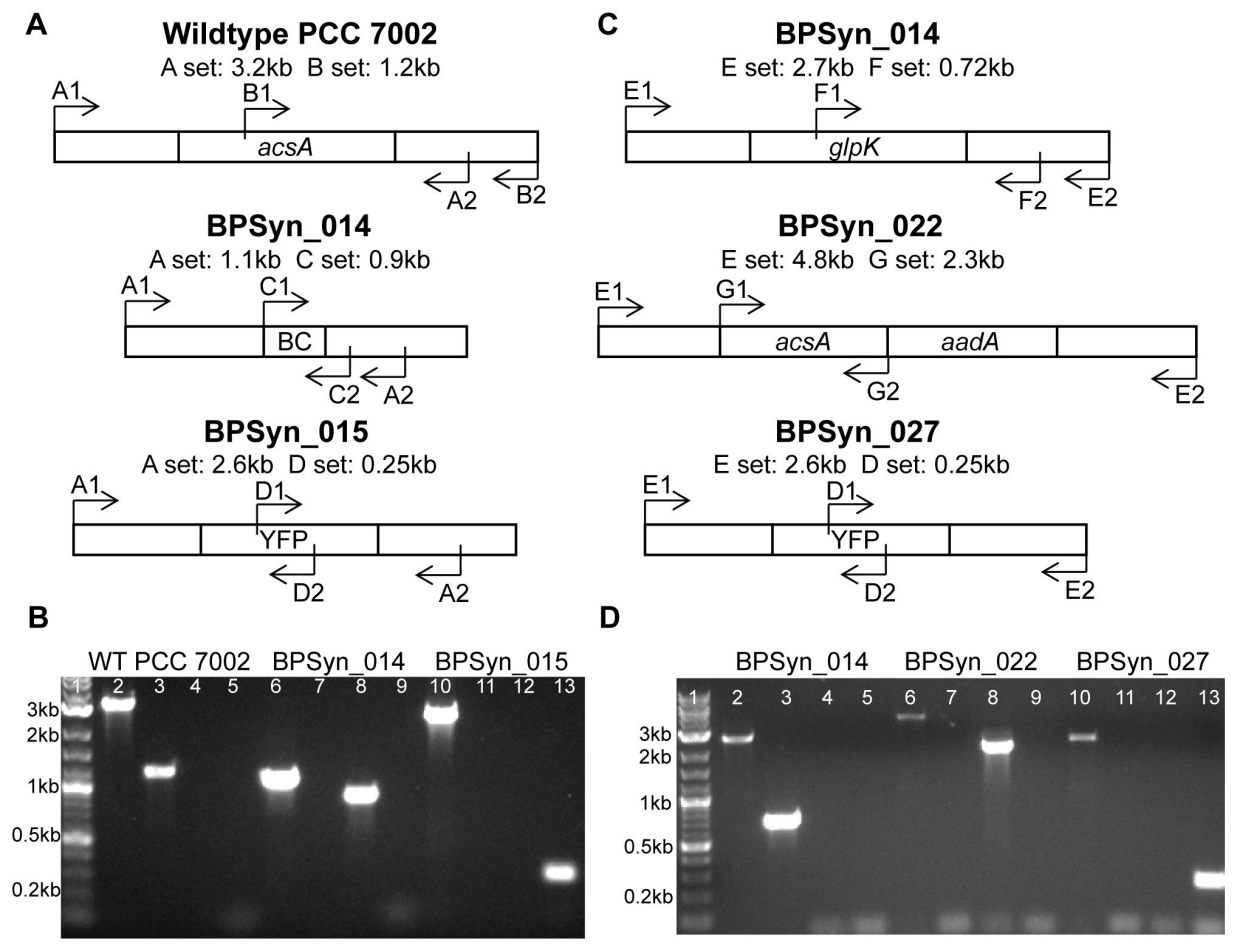
Homozygous mutants were obtained in both the *acsA* and *glpK* loci. (A) Diagram of the primers used to screen for homozygous strains in the *acsA* locus. Primer set A amplifies from the up and down5' and 3' stream flanking regions of *acsA* and should be amplify 3.2 kb, 1.1 kb, and 2.6 kb in fragments for WT 7002, BPSyn_014, and BPSyn_015, respectively. Primer set B amplifies 1.2 kb from inside *acsA* to the region downstream of *acsA*. Primer set C amplifies 0.9 kb from the barcode (BC) region to downstream of *acsA*. Primer set D amplifies a 0.25 kb region internal to YFP. (B) 1% agarose gel showing colony PCR products amplified using the primer sets A, B, C, and D on WT PCC 7002, BPSyn_014, and BPSyn_015. Lane 1 contains a 2-log ladder from New England Biolabs. Lanes 2-5, 6-9, and 10-13 are primers sets A, B, C, and D amplifying WT PCC 7002, BPSyn_014, and BPSyn_015, respectively. (C) Diagram of the primers used to screen for homozygous strains in the *glpK* locus. Primer set E amplifies from the up and down stream5' and 3' flanking regions of *glpK* and should be amplify 2.7 kb, 4.8 kb, and 2.6 kb fragments from BPSyn_014, BPSyn_022, and BPSyn_027, respectively. Primer set F amplifies 0.72 kb from inside *glpK* to the region downstream of *glpK*. Primer set G amplifies a 2.3 kb region of the *acsA* cassette that was introduced into the *glpK* locus. (D) 1% agarose gel showing colony PCR products amplified using the primer sets E, F, G, and D on BPSyn_014, BPSyn_022, and BPSyn_027. Lane 1 contains a 2-log ladder from New England Biolabs. Lanes 2-5, 6-9, and 10-13 are primers sets E, F, G, and D amplifying BPSyn_014, BPSyn_022, and BPSyn_027, respectively.

**Figure 4:**
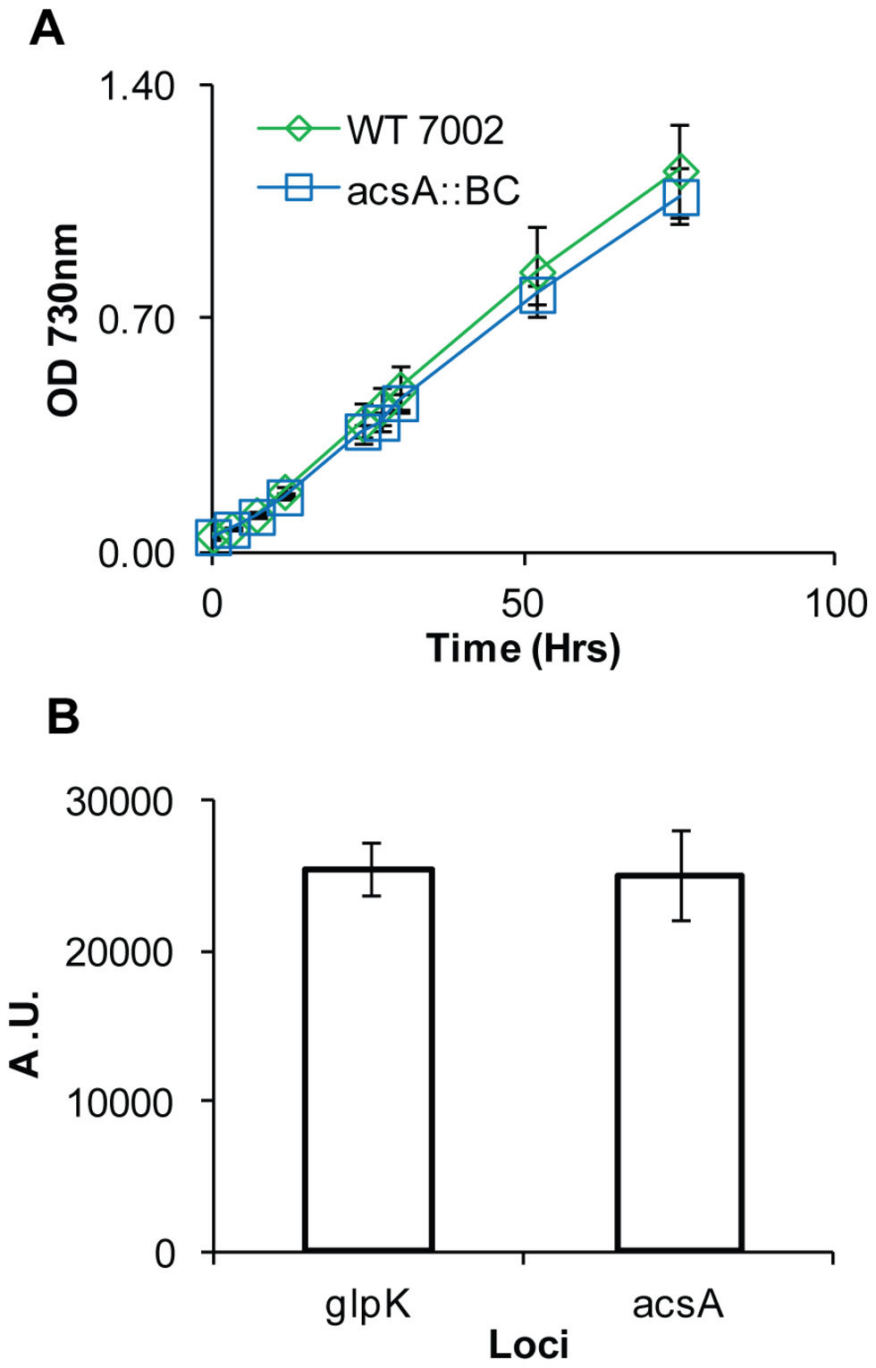
Comparison of *acsA* and *glpK* mutant phenotypes. (A) Growth of wild type PCC 7002 (green diamonds) and BPSyn_014 (blue squares) in Medium A^+^ supplemented with air. (B) Fluorescence measurements of strains BPSyn_015 and BPSyn_027, which contain YFP under the *cpcB* promoter in the *acsA* or *glpK* locus, respectively. Fluorescence is measured using arbitrary units (A.U.) at an excitation of 514 nm and an emission of 527 nm. Data points are the mean of biological triplicates and error bars represent the standard deviation.

Attempts to complement the PCC 7002 *acsA* deletion strain (BPSyn_006) by introducing and expressing *acsA* on the native plasmid pAQ1 or in a chromosomal loci with a kanamycin resistance cassette resulted in heterogeneous strains that could not be made homozygous, and thus could not be used in MIC determination experiments. In order to reuse the *aadA* cassette, a markerless deletion of the native *acsA* locus via counter selection and then reinsert *acsA* and the *aadA* resistance marker into a newly validated neutral insertion site. The *acsA* markerless deletion strain (BPSyn_014) was created by transforming wild type PCC 7002 with a linear DNA fragment to replace *acsA* with a 40 bp barcode (Figure 3A). Transformants were selected on solid medium containing 50 µM acrylate, a low concentration that allows for the growth of heterozygous transformants. Mutants positive for the barcode insertion were streaked onto medium containing 10 mM acrylate. The increase in acrylate concentrations allowed for selection of homozygous clones. Single colonies were demonstrated to be homozygous via colony PCR (Figure 3B). This strain, BPSyn_014, had MIC values identical to BPSyn_006. When compared to wild type PCC 7002, strain BPSyn_014 did not have a significant defect in growth up to 75 h (Figure 4A), but was observed to secrete acetate (370 ± 20 µM for BPSyn_014 vs. none detected for wild type). The markerless deletion allowed for the reuse of the *aadA* selection marker to introduce *acsA* into another locus on the chromosome.

The locus *glpK* was chosen as a neutral insertion site for introduction of genes for complementation of the increased organic acid phenotype. In PCC 7002, *glpK* has a frameshift mutation and is thus considered a pseudogene. Since *glpK* cannot produce a functional protein, it was hypothesized that replacement of *glpK* would not affect physiology. Strain BPSyn_014 was complemented by introducing either (1) a wild type copy of *acsA* or (2) a W49L *acsA* mutant under the native *acsA* promoter along with the *aadA* selection cassette, into the *glpK* locus of the chromosome, resulting in strains BPSyn_022 and BPSyn_026, respectively. The MIC for acrylate and 3-hydroxypropionate for BPSyn_022 were 10µM and 15mM, respectively, similar to wild type PCC 7002 (Table 4). The MIC for acrylate for BPSyn_026 is 7mM, which suggests that the W49L mutant results in only a partial loss of function (Table 4). Additionally, the frequency of acrylate tolerance mutations of BYSyn_022 and BPSyn_026 observed on 50 µM acrylate plates were the same as wildtype PCC 7002.

### Acrylate counter selection can be used for heterologous gene expression

To optimize the counter selection method, the 40 bp barcode sequence used to create strain BPSyn_014 and a linearized plasmid containing His-tagged yellow fluorescent protein (YFP) under the high expression *cpcB* constitutive promoter (pACS_ _p_cpcB_YFP) were transformed into wild type PCC 7002 and selected for on 50, 100, and 500 µM acrylate. After 3 days, colonies containing integrations were only observed on plates containing 50 µM acrylate. The percentage of colonies that were positive for integrations was 49% (n = 42) for the barcode and 30% for YFP (n = 39). A t-test of these data shows no significant difference in integration frequency (p = 0.11) indicating that insertion size had little impact on integration efficiency. Colonies not containing integrations were assumed to be spontaneous mutants of *acsA*. The number of background colonies is consistent with the loss of function mutation frequency previously observed. A homozygous strain for the integration of YFP (BPSyn_015) was obtained by streaking onto plates containing 10 mM acrylate and confirmed by colony PCR (Figure 3B). As expected, strain BPSyn_015 was highly fluorescent (Figure 4B). YFP was successfully purified from BPSyn_015 using Ni-NTA affinity chromatography and visualized with PAGE.

**Figure 5:**
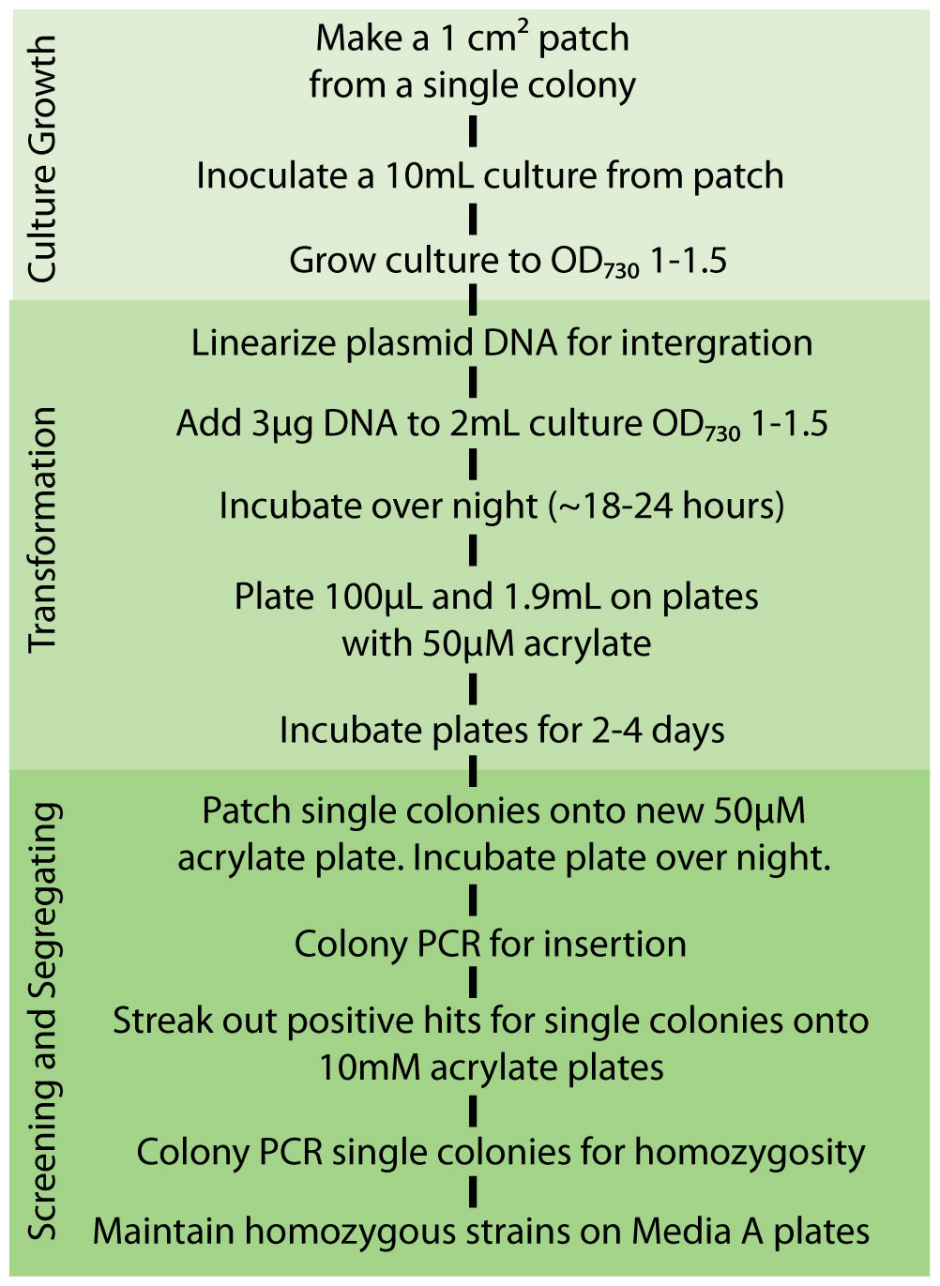
Outline of counter selection protocol based on acrylate sensitivity.

Next, the acrylate counter selection method was used to introduce heterologous genes into other loci. Strain BPSyn_022 (*acsA*::BC *glpK::acsA aadA*) was transformed with the linearized plasmid pGLPK_ _p_cpcB_YFP to replace *acsA* and *aadA* with YFP under the expression of the *cpcB* promoter in the *glpK* locus (Figure 3C). 29% (n=14) of colonies were identified as positive transformants via colony PCR, which is similar to transformations into the *acsA* locus. Positive transformants (BPSyn_027) were verified as homozygous using the same protocol outlined for integration directly into the *acsA* locus (Figure 3D). As expected, YFP was successfully purified from BPSyn_027 and visualized using Ni-NTA affinity chromatography and PAGE. When compared to BPSyn_015, no significant difference was observed in the level of YFP fluorescence (Figure 4B). These results demonstrate that acrylate counter selection can be used to make modifications beyond the native *acsA* locus and that the *glpK* locus has utility as a neutral insertion site. The counter selection methodology is summarized in Figure 5.

## Conclusion

Counter selection is an important tool for genetic manipulation of microorganisms. Counter selection is of particular importance in cyanobacterial systems due to the presence of multiple copies of the chromosome and the difficultly of achieving homozygous strains using antibiotic resistance genes. The results presented in this work describe the basis and methodology for using organic acid sensitivity for counter selection in the cyanobacterium PCC 7002. It was shown that the organic acids acrylate, 3-hydroxypropionate, and propionate cause growth inhibition and that this inhibition can be overcome by loss of the gene *acsA*. Acrylate was further pursued for use in counter selection because of the low concentration required for sensitivity, the dramatic increase in tolerance due to loss of *acsA*, and the potential to be used in other cyanobacteria (e.g. PCC 6803). A method was optimized for introducing genes of interest directly into the *acsA* locus. Loss of *acsA* did not result in a growth defect under the conditions used in this experiment, but did result in an increase in the secretion of acetate by PCC 7002. A neutral site, *glpK*, was identified as a chromosomal locus that is amenable to acrylate counter selection and can be used for expression of heterologous genes. These results suggest that acrylate counter selection could also be used to make markerless deletions or insertions elsewhere on the chromosome. Additionally, a loss of function of *acsA* in PCC 6803 was shown to dramatically increase the tolerance to acrylate, suggesting that this method has utility in multiple species. Application of the method presented in this work will be used in future physiology studies and metabolic engineering efforts.
